# Recurrence and survival after robotic vs laparoscopic liver resection in very-early to early-stage (BCLC 0-A) hepatocellular carcinoma

**DOI:** 10.1007/s00464-025-11553-3

**Published:** 2025-02-04

**Authors:** Lorenzo Bernardi, Emanuele Balzano, Raffaello Roesel, Annamaria Senatore, Daniele Pezzati, Gabriele Catalano, Maria Luisa Garo, Giovanni Tincani, Pietro Majno-Hurst, Davide Ghinolfi, Alessandra Cristaudi

**Affiliations:** 1 Department of Surgery, Lugano Regional Hospital, Ente Ospedaliero Cantonale (EOC), Via Tesserete 46, 6900 Lugano, Switzerland; 2 Hepato-Biliary Surgery and Liver Transplant Division, Azienda Ospedaliera Universitaria Pisana (AOUP), University of Pisa, Via Paradisa, 2, 56124 Pisa, Italy; 3 Campus Bio-medico University of Rome, Rome, Italy; 4 Faculty of Biomedical Science, University of Southern Switzerland (USI), Lugano, Switzerland

**Keywords:** Robotic, Laparoscopic, Liver resection, Hepatocellular carcinoma, Cirrhosis

## Abstract

**Background:**

Robotic (RLR) and laparoscopic liver resection (LLR) for hepatocellular carcinoma (HCC) provide similar short-term outcomes, but data focused on recurrence and survival are still lacking. We hypothesized non-inferior oncologic results of RLR compared to LLR for HCC of stage BCLC 0-A.

**Methods:**

RLRs and LLRs on patients with HCC of stage BCLC 0-A and preserved liver function (Child A or B if cirrhosis) were retrospectively reviewed. Propensity score matching (PSM) was used to mitigate selection bias. The primary endpoints were recurrence-free (RFS) and overall survival (OS); secondary endpoints were incidence, pattern, and treatment of recurrences.

**Results:**

After 1:1 PSM, two groups (RLR = 68; LLR = 68) of patients with similar characteristics, liver function and HCC features were obtained: median age 71-years, males 73.5%, underlying cirrhosis 91.2% (Child A, 96.8%, MELD ≤ 9, 96.0%), portal hypertension 22.1%, single-HCC 90.4%. Two- and 5-year RFS were 78.0 vs 59.0% and 54.0 vs 53.0% (*p* = 0.107), while OS was 97.0 vs 90.0% and 87.0 vs 90.0% (*p* = 0.951) for RLR vs LLR, respectively. Incidence of HCC recurrence was similar (35.3 vs 39.7%; *p* = 0.723). Recurrences developed mostly within the liver (29.4 vs 30.9%; *p* = 1.000) and within 2 years after hepatectomy (19.1 vs 32.4%, *p* = 0.116) in RLR vs LLRs. Curative-intent treatment of recurrences did not differ (liver transplantation 19.6%, redo-resection 15.7%, locoregional treatments 52.9%) except for a tendency toward more redo-resections for recurrences after RLR.

**Conclusions:**

Oncologic outcomes of RLR were not inferior to those of LLR in selected HCC patients of stage BCLC 0-A with underlying cirrhosis. Both techniques guaranteed similar salvageability in case of HCC recurrence.

**Supplementary Information:**

The online version contains supplementary material available at 10.1007/s00464-025-11553-3.

In the field of minimally invasive liver surgery (MILS), both robotic liver resections (RLR) and laparoscopic liver resections (LLR) have been increasingly used for the treatment of patients with early-stage hepatocellular carcinoma (HCC) [1, 2]. Regarding the short-term surgical outcomes, RLR seems to be at least non-inferior to LLR for HCC, while some potential advantages for the robotic approach have been suggested in patients with cirrhosis, supporting its facilitating role over laparoscopy. Alongside to its general peculiarities (such as stable vision, enhanced dexterity and ergonomics), the robotic approach could require shorter Pringle’s duration (or less utilization), less liver mobilization and easier bleeding control, resulting in reduced blood losses and potentially shorter operative time despite the absence of specific robotic instruments for parenchymal dissection [3]. According to a consensus guideline paper recently published, the oncologic efficacy of RLR compared to that of LLR (recurrence and survival) has to be further investigated [4]. Moreover, only few small studies looked at long-term outcomes in cohorts with high rates of cirrhosis [5–7]. Several other series included a low percentage of cirrhotic patients or did not state the rates of cirrhosis at all, making it difficult to extrapolate conclusions [8–13]. The aim of this study was to demonstrate the non-inferior oncologic outcomes of RLR compared to LLR for HCC of stage BCLC 0-A in a population with high prevalence of cirrhosis.

## Methods

### Study design and setting

This study was conducted in compliance with the STROCSS guidelines for reporting cohort studies in surgery [14]. This was an observational, retrospective, cohort study including patients underwent minimally invasive liver resection (either robotic or laparoscopic) at the Hepatobiliary surgery and liver transplant division, University of Pisa Medical School Hospital, Italy and the Hepatobiliary surgery Unit, Lugano Regional Hospital, Switzerland. Both centers belong to tertiary care hospitals and have extensive experience in liver surgery.

### Ethical approval and Informed Consent Statement

The study was approved by the regional ethical committees of the institutions participating in the study (Institutional Review Board of the University Hospital of Pisa, Italy, and Comitato Etico Cantonale del Ticino, Lugano, Switzerland, project-ID 2023-01022 CE 4377), and was registered at ClinicalTrials.gov (Registration Number: NCT06496113). All patients were provided with an informed consent (Pisa) or non-objection letter (Lugano).

### Participants

#### Inclusion criteria

Patients underwent robotic and laparoscopic resections for histology-confirmed HCC of stage BCLC 0-A in the period January 2014 to March 2023, patients > 18 years old, with preserved liver function (Child A or B if cirrhosis) were included. Pre-treated HCC patients (i.e., previous liver surgery or locoregional treatments) were not excluded from the analysis.

#### Exclusion criteria

Hand-assisted procedures, non-elective liver resections, intraoperative switch to locoregional therapies like radiofrequency or microwave ablation, non-histologically confirmed HCC, mixed hepato-cholangiocarcinoma were excluded.

### Data source and measurements

Patients were divided in two groups according to the surgical approach (RLR or LLR). Data collected included patient-features, liver function (presence of underlying cirrhosis, Child–Pugh status, MELD score) and tumor details (number of HCC and size), surgical procedures. The diagnosis of cirrhosis was based on clinical, biochemical, radiologic, and pathological data. Portal hypertension was defined according to the ESMO guidelines [15]. Resections were defined according to the Brisbane nomenclature [16]. All procedures were stratified as per level of difficulty according to the Iwate criteria [17]. In case of patients with multiple liver resections, the resection with the highest score was used to establish the difficulty index. The principles of parenchymal sparing surgery (PSS) were systematically adopted [18].

HCC diagnosis and postoperative follow-up were conducted according to recent guidelines [15]. Minimum follow-up accepted was of 3 months postoperatively. Recurrence was dated at the first HCC detection on follow-up imaging. Survival was censored at death or latest follow-up. Liver transplantation (salvage-LT) was considered in case of HCC recurrence or liver function decompensation when indicated [19–23].

### Indications to surgery

The allocation to surgical treatment was decided at multidisciplinary team (MDT) discussion based on the BCLC staging system and treatment algorithm [24]. With occasional exceptions MILS could be performed in selected Child B patients and in the presence of portal hypertension for resections that involved minimal parenchymal sacrifice or in selected BCLA A patients with 2 or three nodules when transplantation was deemed not indicated and surgery particularly non-invasive (i.e., older patients with general comorbidities, history of recent alcohol use, refusal to participate in a transplant program, as well as small superficial nodules easily accessible with MILS) [25, 26]. The indications for RLR and LLR for HCC were in principle the same. The choice of the approach (RLR, LLR) relied basically on the availability of the robotic platform (1 session every 1 or 2 weeks), when the robot was not available patients were considered for LLR (freely available up to 2 times per week). All the surgical procedures were performed by senior hepatobiliary surgeons, the surgical technique for both RLR and LLR and the details of MILS program implementation in both centers were described elsewhere. Briefly, RLR and LLR were introduced at the same time in one center (Pisa), while RLR followed LLR in the other (Lugano). Importantly, both approaches were performed concurrently in the study period and LLR was never abandoned, due to the restricted availability of the robot in both institutions [3, 27, 28].

### Outcomes

The primary outcomes of the study were RFS and OS. Secondary outcomes were the incidence and pattern of recurrences (intrahepatic, extrahepatic, sites of recurrence), and the treatment of recurrences. Surgical-related outcomes (within 90 days after surgery) such as postoperative morbidity (according to Clavien-Dindo classification of surgical complications), comprehensive complication index (CCI), postoperative biliary leak and post-hepatectomy liver failure rates (both according to ISGLS definitions), hospital stay, and surgical-related mortality were also collected [29–31].

### Statistical analysis

Descriptive statistics are reported as relative frequencies and percentages for categorical and dichotomous variables and as median and IQR for quantitative variables. Comparisons between LLR and RLR were performed using Fisher’s exact test. For continuous variables, the U-Mann–Whitney test was applied after checking the distribution of variables with the Shapiro–Wilk test. To adjust for confounders, 1:1 propensity score matching (PSM) was applied. Propensity scores were estimated using a logistic regression model that used presence of cirrhosis (no vs yes), portal hypertension (no vs yes), previous HCC treatment (no vs yes), number of nodules (single vs multiple), and Iwate score as predictors. The selection of predictors was based on the literature as well as expert opinion and statistical inference. For PSM, we used the nearest neighbor matching method without replacement of patients and without using a caliper. We then checked whether the covariates in each group were balanced using the above statistical tests. Recurrence-free survival (RFS) and Overall survival (OS) were analyzed using the Kaplan–Meier method. Comparison between the two groups was performed by log-rank test.

The statistical significance level for all statistical analyzes was set at 5% (*p* < 0.05). Borderline statistically significant differences were examined according to the variability of the variables and clinical considerations. Statistical analysis was performed using STATA18 (StataCorp., College Station, TX, USA).

## Results

### Participants

#### Before PSM

**Fig. 1:**
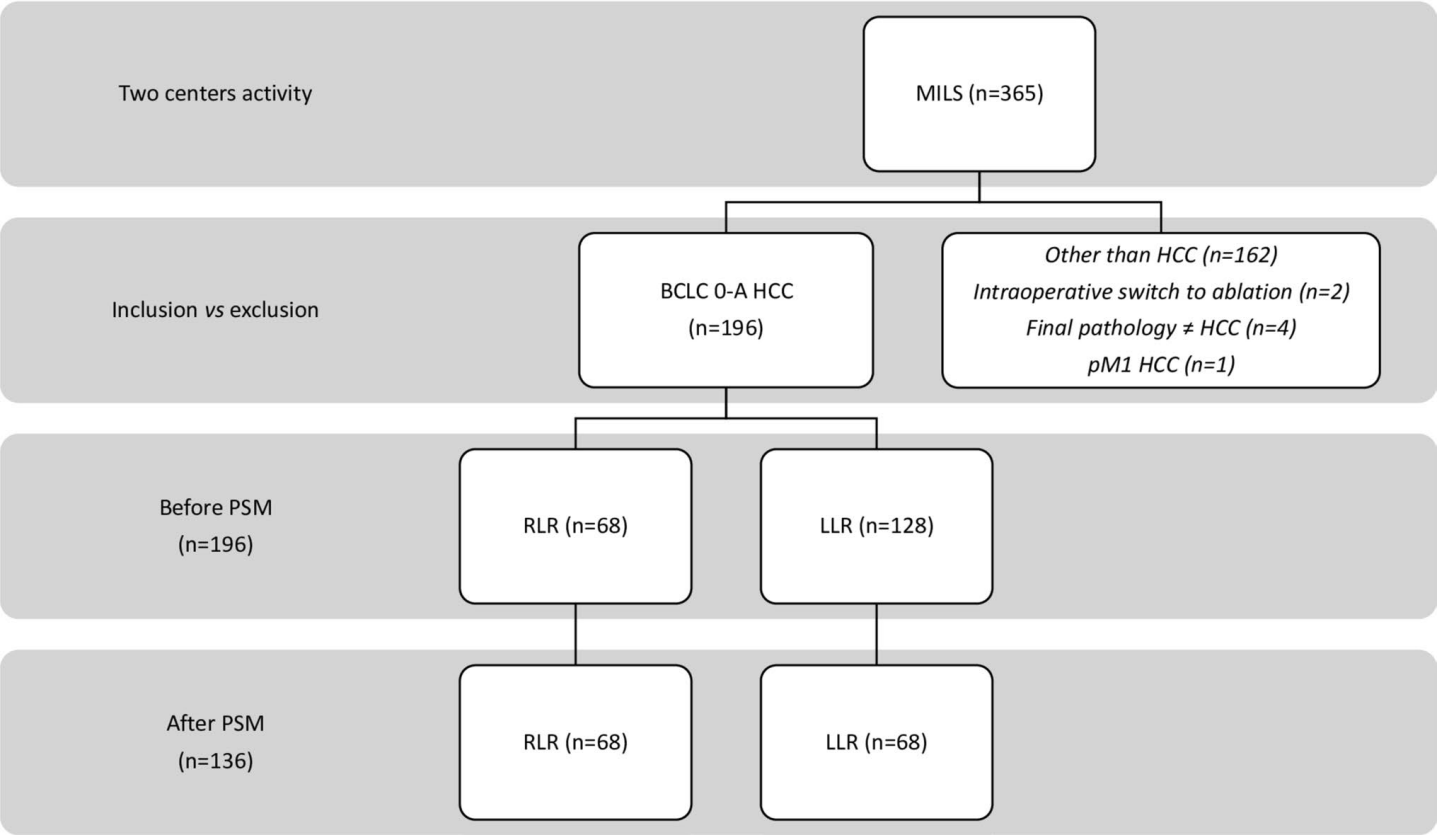
Flowchart of the study

A total of 365 MILS patients potentially eligible were screened. Among those, 196 patients underwent MILS for HCC of stage BCLC 0-A (LLR = 128; RLR = 68) were confirmed eligible and included in the study (shown in Fig. 1). The two populations (LLR and RLR) were similar except for MELD value 9 or less, and portal hypertension, the latter being more frequent in RLR group [LLR 11 (8.6%) vs RLR 19 (27.9%); *p* = 0.001]. Baseline characteristics of the two groups before the PSM are shown in Table 1.

**Table 1:** Baseline characteristics of the population and in robotic vs laparoscopic liver resections groups

	Before PSM				After PSM			
	MILS (*n* = 196)	LLR (*n* = 128)	RLR (*n* = 68)	*p*-value	MILS (*n* = 136)	LLR (*n* = 68)	RLR (*n* = 68)	*p*-value
Age, median (IQR)	71 (62–76)	71 (62–77)	70 (62–75)	0.503	71 (62–76)	71 (62–76)	70 (62–75)	0.613
Gender, n (%)								
Male	145 (74.0)	97 (75.8)	48 (70.6)	0.495	100 (73.5)	52 (76.5)	48 (70.6)	0.560
Female	51 (26.0)	31 (24.2)	20 (29.4)		36 (26.5)	16 (23.5)	20 (29.4)	
BMI, median (IQR)	25 (23–27)	25 (23–27)	25.4 (23–27.7)	0.496	25 (23–27)	25 (23–27)	25.4 (23–27.7)	0.582
ASA, median (Range)*	3 (1–4)	3 (1–4)	3 (1–4)	0.458	3 (1–4)	3 (1–4)	3 (1–4)	0.098
Previous Abdominal Surgery, n (%)	96 (49.0)	62 (48.4)	34 (50.0)	0.881	68 (50.0)	34 (50.0)	34 (50.0)	1.000
Previous liver surgery, n (%)	17 (10.1)	12 (9.4)	5 (7.4)	0.792	13 (9.6)	8 (11.8)	5 (7.4)	0.561
Previous local treatment, n (%)								
None	160 (81.6)	100 (78.1)	60 (88.2)	0.669	110 (80.9)	50 (73.5)	60 (88.2)	0.207
RFA	17 (8.7)	13 (10.2)	4 (5.9)		13 (9.6)	9 (13.2)	4 (5.9)	
TACE	15 (7.7)	11 (8.6)	4 (5.9)		10 (7.4)	6 (8.8)	4 (5.9)	
TARE	1 (0.5)	1 (0.8)	0 (0.0)		1 (0.7)	1 (1.5)	0 (0.0)	
RFA + TACE	2 (1.0)	2 (1.6)	0 (0.0)		1 (0.7)	1 (1.5)	0 (0.0)	
Cirrhosis, n (%)	173 (88.3)	111 (86.7)	62 (91.2)	0.485	124 (91.2)	62 (91.2)	62 (91.2)	1.000
Child Pugh, n (%)								
Child A	168 (97.1)	110 (99.1)	58 (93.6)	0.056	120 (96.8)	62 (100.0)	58 (93.6)	0.119
Child B	5 (2.9)	1 (0.9)	4 (6.5)		4 (3.2)	0 (0.0)	4 (6.5)	
MELD, n (%)								
≤ 9	168 (97.1)	111 (100.0)	57 (91.9)	**0.005**	119 (96.0)	62 (100.0)	57 (91.9)	0.057
> 9	5 (2.89)	0 (0.0)	5 (8.1)		5 (4.0)	0 (0.0)	5 (8.1)	
Underlying liver disease, n (%)								
None	19 (9.7)	13 (10.2)	6 (8.8)	0.247	10 (7.4)	4 (5.9)	6 (8.8)	0.633
Biliary Cirrhosis	2 (1)	2 (1.6)	0 (0.0)		1 (0.7)	1 (1.5)	0 (0.0)	
Cryptogenic	6 (3.1)	1 (0.8)	5 (7.4)		6 (4.4)	1 (1.5)	5 (7.4)	
HBV	19 (9.7)	11 (8.6)	8 (11.8)		15 (11.0)	7 (10.3)	8 (11.8)	
HCV	94 (48.0)	63 (49.2)	31 (45.6)		66 (48.5)	35 (51.5)	31 (45.6)	
HCV HBV	1 (0.5)	1 (0.8)	0 (0.0)		1 (0.7)	1 (1.5)	0 (0.0)	
HCV/OH	1 (0.5)	0 (0.0)	1 (1.5)		1 (0.7)	0 (0.0)	1 (1.5)	
Hemochromatosis	6 (3.1)	5 (3.9)	1 (1.5)		3 (2.2)	2 (2.9)	1 (1.5)	
NAFLD	3 (1.5)	2 (1.6)	1 (1.5)		2 (1.5)	1 (1.5)	1 (1.5)	
NASH	24 (12.2)	18 (14.1)	6 (8.8)		16 (11.8)	10 (14.7)	6 (8.8)	
OH	21 (10.7)	12 (9.4)	9 (13.2)		15 (11.0)	6 (8.8)	9 (13.2)	
Portal hypertension, n (%)	30 (15.3)	11 (8.6)	19 (27.9)	**0.001**	30 (22.1)	11 (16.2)	19 (27.9)	0.147
Nodules, n (%)								
Single	171 (87.2)	111 (86.7)	60 (88.2)	0.826	123 (90.4)	63 (92.7)	60 (88.2)	0.561
Multiple	25 (12.8)	17 (13.3)	8 (11.8)		13 (9.6)	5 (7.4)	8 (11.8)	
Size, median (IQR)	25 (18.5–35)	25.5 (20–35)	23 (18–30)	0.079	24 (18–30)	25 (17.5–30.5)	23 (18–30)	0.403
Stage BCLC-0, n (%)	70 (35.7)	42 (32.8)	28 (41.2)	0.275	54 (39.7)	26 (38.2)	28 (41.2)	0.861
Stage BCLC-A, n (%)	126 (64.3)	86 (67.2)	40 (58.8)	0.275	82 (60.3)	42 (61.8)	40 (58.8)	0.861
Stage BCLC-A with single HCC, n (%)	107 (54.6)	75 (58.6)	32 (47.1)	0.134	71 (52.2)	39 (57.4)	32 (47.1)	0.303

*MILS* minimally invasive liver surgery; *RLR* robotic liver resection; *LLR* laparoscopic live resection; *RFA* radiofrequency ablation; *TACE* trans arterial chemoembolization; *TARE* trans arterial radioembolization; *MELD* model for end stage liver disease; *NAFLD* non-alcoholic fatty liver disease; *NASH* non-alcoholic steatohepatitis; *OH* alcohol; *BCLC* Barcelona Clinic Liver Cancer

Bold characters highlight statistical significance

^*^Range (min–max) instead of IQR to better describe variability

#### After PSM

After 1:1 matching, 136 patients of similar characteristics, liver function and HCC features were obtained (LLR = 68; RLR = 68) (Fig. 1). The median age (IQR) was of 71 (62–76) years. One-hundred patients (73.5%) were males. Median BMI (IQR) was 25.0 kg/m2 (23.0–27.0). One-hundred-twenty-four patients (91.2%) had underlying liver cirrhosis, of Child–Pugh class A in 96.8% of cases. MELD was 9 or less in 119 patients (96.0%). Cirrhosis was mostly related to HCV infection (66 patients, 48.5%). Portal hypertension was present in 30 patients (22.1%). HCC consisted of a single nodule in 123 patients (90.4%), the remainder of patients (9.6%) had multiple nodules (up to 3). Median size of HCC (IQR) was 24.0 mm (18.0–30.0). Fifty-four patients (39.7%) were classed as BCLC stage 0; while 82 (60.3%) were BCLC A. Specifically, 71 patients (52.2%) were classed BCLC A with a single tumor. The characteristics of the two groups after PSM are shown in Table 1.

### Procedures and surgery-related outcomes

#### Before PSM

**Fig. 2:**
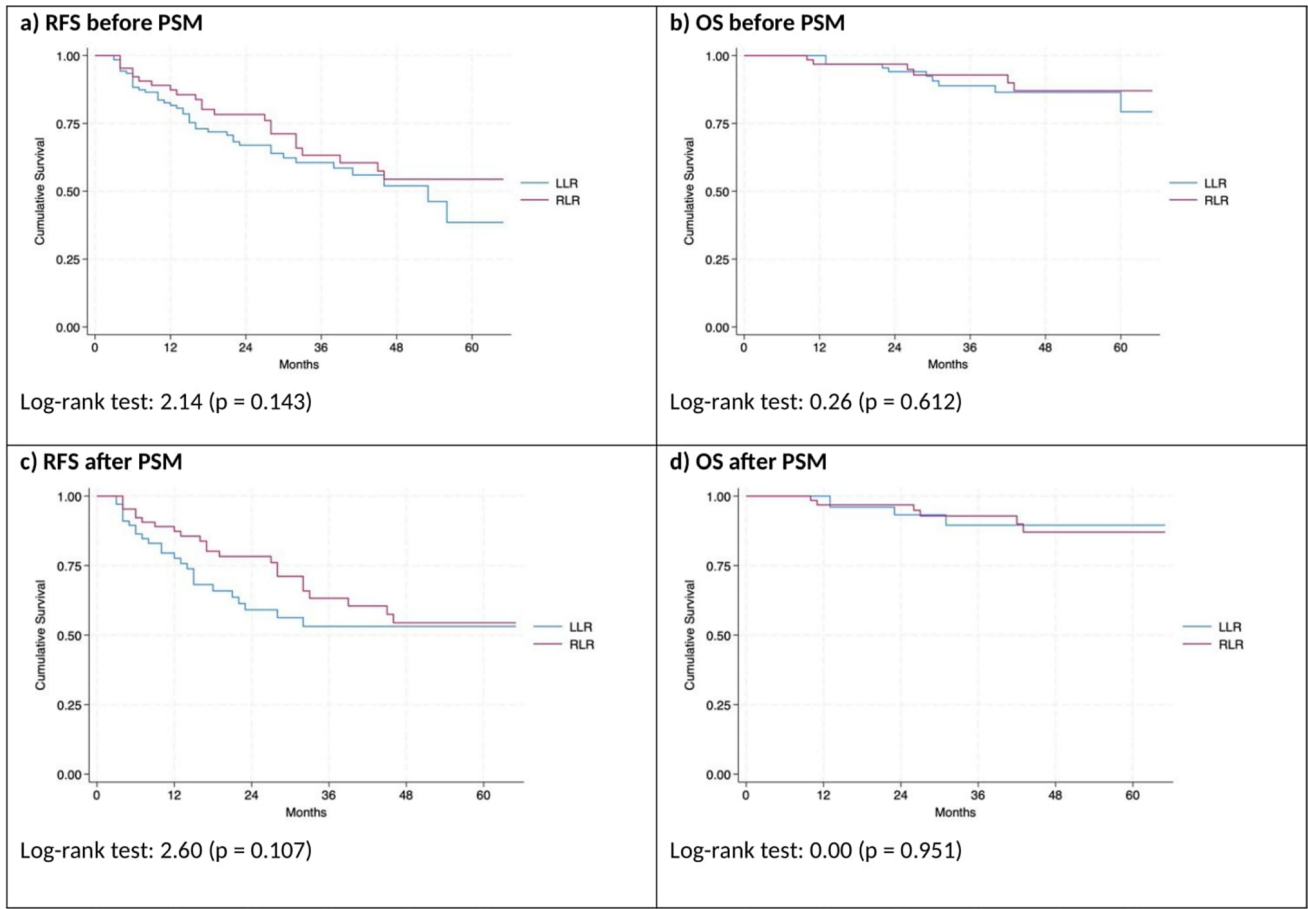
Kaplan–Meier curve for Recurrence-Free Survival (RFS) and Overall Survival (OS) before and after PSM in total sample

One-hundred-fifty-three patients out of 196 (78.1%) underwent non-anatomic resections [LLR 92 (71.9%) vs RLR 61 (89.7%); (*p* = 0.048)]. Compared to RLR, LLR group consisted of more complex resections according to the Iwate criteria (*p* = 0.017). The hilar clamping (Pringle maneuver) was used more often and lasted longer in LLR than in RLR. No difference was observed in operative and postoperative outcomes of LLR and RLR, except less intraoperative blood loss in RLR group (Fig. 2).

#### After PSM

One-hundred-nineteen patients out of 136 (87.5%) underwent non-anatomic resections. Iwate score (median) of MILS was 4.0 and was well balanced between the two groups. Pringle maneuver was still more used in LLR and lasted longer than in RLR (*p* < 0.001) and intraoperative blood loss (IQR) was higher in LLR vs RLR (*p* = 0.013). Other surgical outcomes were similar between RLR and LLR, except the median CCI (IQR) which was lower in RLR (*p* = 0.034). Microscopically complete resection (R0 margin) rates were similar in the two groups (95.6 vs 92.6% in LLR vs RLR; *p* = 0.466). The surgical outcomes and pathology data before and after the PSM data were reported in Table 2.

**Table 2:** Procedures, surgical outcomes, and pathology data (overall and after robotic vs laparoscopic liver resections)

	Before PSM				After PSM			
	MILS (*n* = 196)	LLR (*n* = 128)	RLR (*n* = 68)	*p*-value	MILS (*n* = 136)	LLR (*n* = 68)	RLR (n = 68)	*p*-value
Procedures, n (%)								
Wedge/non-anatomical resection	153 (78.1)	92 (71.9)	61 (89.7)	**0.048**	119 (87.5)	58 (85.3)	61 (89.7)	0.797
Sub-segmentectomy	11 (5.6)	8 (6.2)	3 (4.4)		6 (4.4)	3 (4.4)	3 (4.4)	
Segmentectomy	18 (9.2)	15 (11.7)	3 (4.4)		9 (6.6)	6 (8.8)	3 (4.4)	
Bi-segmentectomy	12 (6.1)	11 (8.6)	1 (1.5)		2 (1.5)	1 (1.5)	1 (1.5)	
Right hemi-hepatectomy	2 (1.0)	2 (1.6)	0 (0.0)		0 (0.0)	0 (0.0)	0 (0.0)	
Associated local treatment	9 (4.6)	9 (7.0)	0 (0.0)		4 (2.9)	4 (5.9)	0 (0.0)	
IWATE score, median (IQR)	4 (2–6)	4 (2–6)	4 (2–5)	0.475	4.0 (2–5.5)	3.5 (2–6)	4 (2–5)	0.857
IWATE level, n (%)								
Low	91 (46.4)	59 (46.1)	32 (47.1)	**0.017**	66 (48.5)	34 (50.0)	32 (47.1)	0.375
Intermediate	74 (37.8)	42 (32.8)	32 (47.1)		58 (42.6)	26 (38.2)	32 (47.1)	
Advanced	23 (11.7)	19 (14.8)	4 (5.9)		12 (8.8)	8 (11.8)	4 (5.9)	
Expert	8 (4.1)	8 (6.2)	0 (0.0)		0 (0.0)	0 (0.0)	0 (0.0)	
Pringle’s maneuver, n (%)	78 (39.8)	63 (49.2)	15 (22.1)	** < 0.001**	45 (33.1)	30 (44.1)	15 (22.1)	**0.010**
Pringle’s duration (min), median (IQR)	30 (0–50)	39.5 (24–60)	0 (0–20)	** < 0.001**	12 (0–45)	30 (15–52)	0 (0–20)	** < 0.001**
Operative time (min), median (IQR)	225 (170–291.5)	237.5 (175–297.5)	210 (166–274.5)	0.180	222.5 (165–279.5)	225 (165–280)	210 (166–274.5)	0.728
Conversion, n (%)	19 (9.7)	14 (10.9)	5 (7.4)	0.613	12 (8.8)	7 (10.3)	5 (7.4)	0.764
Intraoperative transfusion, n (%)	4 (2.0)	2 (1.6)	2 (2.9)	0.611	3 (2.2)	1 (1.5)	2 (2.9)	1.000
Blood loss (ml), median (IQR)	200 (100–200)	200 (100–300)	100 (100–200)	** < 0.001**	150 (100–200)	200 (100–300)	100 (100–200)	**0.013**
LOS (days), median (IQR)	6 (5–8)	6 (5–8)	6 (5–7)	0.709	6 (4–8)	5.5 (4–8)	6 (5–7)	0.880
90-day morbidity (Clavien-Dindo), n (%)	51 (26.0)	38 (29.7)	13 (19.1)	0.125	35 (25.7)	22 (32.4)	13 (19.1)	0.116
90-day major morbidity (Clavien-Dindo), n (%)	7 (3.6)	5 (3.9)	2 (2.9)	1.000	5 (3.7)	3 (4.4)	2 (2.9)	1.000
CCI, median (IQR)	0 (0–8.7)	0 (0–8.7)	0 (0–0)	0.069	0 (0–8.7)	0 (0–8.7)	0 (0–0)	**0.034**
Biliary fistula (ISGLS)*, n (%)	5 (2.6)	5 (3.9)	0 (0.0)	0.166	1 (0.7)	1 (1.5)	0 (0.0)	1.000
PHLF (ISGLS), n (%)	15 (7.7)	12 (9.4)	3 (4.4)	0.268	11 (8.1)	8 (11.8)	3 (4.4)	0.207
PHLF grade, n (%)								
A	8 (4.1)	6 (4.7)	2 (2.9)	1.000	7 (5.1)	5 (7.4)	2 (2.9)	1.000
B	7 (3.6)	6 (4.7)	1 (1.5)		4 (2.9)	3 (4.4)	1 (1.5)	
90-day mortality, n (%)	0 (0.0)	0 (0.0)	0 (0.0)	NA	0 (0.0)	0 (0.0)	0 (0.0)	NA
Pathology data								
Microvascular invasion, n (%)	64 (32.6)	50 (39.0)	14 (20.6)	** < 0.001**	40 (29.4)	26 (38.2)	14 (20.6)	0.056
Satellite nodules, n (%)	10 (5.1)	8 (6.2)	2 (3.0)	0.050	6 (4.4)	4 (5.9)	2 (3.0)	0.410
Grade, median (IQR)	2 (1–3)	2 (1–3)	2 (2–3)	1.000	2 (1–3)	2 (1–3)	2 (2–3)	0.862
Resection Margin, n (%)								
R0	186 (94.9)	123 (96.1)	63 (92.6)	0.297	128 (94.1)	65 (95.6)	63 (92.6)	0.466
R1	10 (5.1)	5 (3.9)	5 (7.4)		8 (5.9)	3 (4.4)	5 (7.4)	

*All grade A

Bold characters highlight statistical significance

*MILS* minimally invasive liver surgery; *RLR* robotic liver resection; *LLR* laparoscopic live resection; *LOS* Length of hospital stay; *CCI* comprehensive complication index; *ISGLS* International study group of liver surgery; *PHLF* post-hepatectomy liver failure

### Long-term outcomes

### Before PSM

No difference was observed in 2- or 5-year RFS and OS. After a median follow-up of 28 months, 18 patients (9.2%) died [LLR 11 (8.6%) vs RLR 7 (10.3%); *p* = 0.796]. The incidence, timing (proportion of patients with early recurrences within < 1 and < 2 years after hepatectomy) and pattern of recurrences were also similar in LLR vs RLR. Regarding the treatment of recurrences, LLR and RLR showed similar salvageability with a similar proportion of patients who received a curative-intent treatment for recurrence (i.e., salvage-LT or redo-hepatectomy) or other treatments (locoregional, systemic, or best supportive care). The long-term outcomes of LLR vs RLR and the treatment of recurrences before the PSM are detailed in Tables 3 and 4.

**Table 3:** Long-term outcomes (recurrences and survival), overall and after robotic *vs* laparoscopic liver resections

	Before PSM				After PSM			
	MILS (*n* = 196)	LLR (*n* = 128)	RLR (*n* = 68)	*p*-value	MILS (*n* = 136)	LLR (*n* = 68)	RLR (*n* = 68)	*p*-value
Follow-up (months), median (IQR)	28 (13–46)				29 (15–48)			
Death, n (%)	18 (9.2)	11 (8.6)	7 (10.3)	0.796	12 (8.8)	5 (7.4)	7 (10.3)	0.764
Recurrence, n (%)	69 (35.2)	45 (35.2)	24 (35.3)	1.000	51 (37.5)	27 (39.7)	24 (35.3)	0.723
Median RFS (months), median (IQR)	20 (9–34)	17 (8–34)	24 (12–39)	0.130	19 (9–34)	15 (8–33)	24 (12–39)	0.117
Recurrence within 1 year after hepatectomy, n (%)	28 (14.3)	21 (16.4)	7 (10.3)	0.289	21 (15.4)	14 (20.6)	7 (10.3)	0.153
Recurrence within 2 years after hepatectomy, n (%)	46 (23.5)	33 (25.8)	13 (19.1)	0.376	35 (25.7)	22 (32.4)	13 (19.1)	0.116
Liver recurrence only, n (%)	53 (27.0)	33 (25.8)	20 (29.4)	0.351	41 (30.1)	21 (30.9)	20 (29.4)	1.000
Extrahepatic recurrence only, n (%)	5 (2.6)	4 (3.1)	1 (1.5)	0.660	2 (1.5)	1 (1.5)	1 (1.5)	1.000
Both (intra- and extra-hepatic), n (%)	11 (5.6)	8 (6.3)	3 (4.4)	0.751	8 (5.9)	5 (7.4)	3 (4.4)	0.718
Site of liver recurrence, n (%)								
At surgical margin	7 (3.6)	6 (4.7)	1 (1.5)	0.425	5 (3.7)	4 (5.9)	1 (1.5)	0.366
Other intrahepatic	47 (24.0)	29 (22.7)	18 (26.5)	0.600	36 (26.5)	18 (26.5)	18 (26.5)	1.000
Both	10 (5.1)	6 (4.7)	4 (5.9)	0.741	8 (5.9)	4 (5.9)	4 (5.9)	1.000
Site of extra-hepatic recurrence, n (%)								
Lung	5 (2.6)	5 (3.9)	0 (0.0)	0.166	3 (2.2)	3 (4.4)	0 (0.0)	0.244
Bone	4 (2.0)	3 (2.3)	1 (1.5)	1.000	4 (2.9)	3 (4.4)	1 (1.5)	0.619
Abdominal/peritoneal	5 (2.6)	3 (2.3)	2 (2.9)	1.000	3 (2.2)	1 (1.5)	2 (2.9)	1.000
Lymph nodes	3 (1.5)	3 (2.3)	0 (0.0)	0.553	0 (0.0)	0 (0.0)	0 (0.0)	NA
Adrenal gland	1 (0.5)	0 (0.0)	1 (1.5)	0.347	1 (0.7)	0 (0.0)	1 (1.5)	1.000
Brain	1 (0.5)	1 (0.8)	0 (0.0)	1.000	1 (0.7)	1 (1.5)	0 (0.0)	1.000
Multiple extrahepatic (> 1 site)	3 (1.5)	3 (2.3)	0 (0.0)	0.553	2 (1.5)	2 (2.9)	0 (0.0)	0.496
Survival estimated 2-year RFS (95%CI)	0.71 (0.63–0.78)	0.67(0.57–0.75)	0.78 (0.65–0.87)	0.143	0.69 (0.60–0.77)	0.59 (0.45–0.71)	0.78 (0.65–0.87)	0.107
Survival estimated 5-year RFS (95%CI)	0.48 (0.37–0.58)	0.39 (0.20–0.57)	0.54 (0.38–0.68)		0.53 (0.41–0.63)	0.53 (0.38–0.66)	0.54 (0.38–0.68)	
Survival estimated 2-year OS (95%CI)	0.95 (0.90–0.98)	0.94 (0.86–0.98)	0.97 (0.88–0.99)	0.612	0.95 (0.89–0.98)	0.90 (0.80–0.98)	0.97 (0.88–0.99)	0.951
Survival estimated 5-year OS (95%CI)	0.83 (0.73–0.90)	0.79 (0.58–0.91)	0.87 (0.73–0.94)		0.88 (0.77–0.93)	0.90 (0.73–0.96)	0.87 (0.73–0.94)	

Bold characters highlight statistical significance

*NE* not estimated, *OS* overall survival; *RFS* recurrence-free survival. *MILS* minimally invasive liver surgery; *RLR* robotic liver resection; *LLR* laparoscopic live resection

**Table 4:** Treatment of recurrences, overall and after robotic *vs* laparoscopic liver resections

Recurrence treatment, n (%)	Before PSM				After PSM			
	MILS (*n* = 69)	LLR (*n* = 45)	RLR (*n* = 24)	*p*-value	MILS (*n* = 51)	LLR (*n* = 27)	RLR (*n* = 24)	*p*-value
Salvage liver transplant	12 **(**17.4)	8 (17.8)	4 (16.7)	1.000	10 (19.6)	6 (22.2)	4 (16.7)	0.744
Redo-hepatectomy	12 **(**17.4)	5 (11.1)	7 (29.2)	0.114	8 (15.7)	1 (3.7)	7 (29.2)	0.062
Locoregional treatments (all)	33 (47.8)	20 (44.4)	13 (54.2)	0.444	27 (52.9)	14 (51.9)	13 (54.2)	0.782
RFA	22 (31.9)	14 (31.1)	8 (33.3)	0.789	16 (31.4)	8 (29.6)	8 (33.3)	0.767
TACE	17 (24.6)	9 (20.0)	8 (33.3)	0.239	15 (29.4)	7 (25.9)	8 (33.3)	0.548
TARE	2 (2.9)	2 (4.4)	0 (0.0)	0.546	1 (2.0)	1 (3.7)	0 (0.0)	1.000
Systemic Treatment	20 (29.0)	14 (31.1)	6 (25.0)	0.781	12 (23.5)	6 (22.2)	6 (25.0)	1.000
Other/BSC/Not specified	8 (11.6)	5 (11.1)	3 (12.5)	1.000	6 (11.8)	3 (11.1)	3 (12.5)	1.000
Patients having multiple treatments, n (%)	26 (37.7)	16 (35.6)	10 (41.7)	0.602	20 (39.2)	10 (37.0)	10 (41.7)	0.774

*MILS* minimally invasive liver surgery; *RLR* robotic liver resection; *LLR* laparoscopic live resection; *RFA* radiofrequency ablation; *TACE* trans arterial chemoembolization; *TARE* trans arterial radioembolization; *BSC* best supportive care

### After PSM

#### Recurrence-free survival (RFS) and overall survival (OS)

Estimated 2-year and 5-year RFS (95%CI) were 69.0% [LLR 59.0% vs RLR 78.0%; *p* = 0.107], and 53.0% [LLR 53.0% vs RLR 54.0%; *p* = 0.107]. Estimated 2-year and 5-year OS (95%CI) were 95.0% [LLR 90.0% vs RLR 97.0%; *p* = 0.951] and 88.0% [LLR 90.0% vs RLR 87.0%; *p* = 0.951], respectively (Table 3). Twelve patients died (8.8%) [LLR 5 (7.4%) vs RLR 7 (10.3%); *p* = 0.764] after a median follow-up of 29.0 months.

#### Incidence, timing, and pattern of recurrence

Fifty-one patients of 136 included in the PSM (37.5%) had HCC recurrence [LLR 27 (39.7%) vs RLR 24 (35.3%); *p* = 0.723]. Twenty-one patients (15.4%) [LLR 14 (20.6%) vs RLR 7 (10.3%); *p* = 0.153] developed HCC recurrence within 1 year after liver resection, while and 35 (25.7%) [LLR 22 (32.4%) vs RLR 13 (19.1%); *p* = 0.116] within 2 years after liver resection (shown in Table 3). No significant difference in OS was observed in such patients in LLR vs RLR groups (Supplementary Material, Figure S2).

Overall, 41 patients (30.1%) developed intra-hepatic recurrence only [LLR 21 (30.9%) vs RLR 20 (29.4%) *p* = 1.000]. Two patients (1.5%) developed extra-hepatic recurrence only (1 in each group) and 8 (5.9%) both intra- and extra-hepatic recurrence [LLR 5 (7.4%) vs RLR 3 (4.4%); *p* = 0.718]. Regarding intra-hepatic recurrences (isolated, or both intra- and extra-hepatic), these were at the surgical margin in 5 patients (3.7%) [LLR 4 (5.9%) vs RLR 1 (1.5%); *p* = 0.366]; distant within the liver in 36 (26.5%) or in both sites in 8 patients (5.9%) equally distributed in LLR and RLR. With decreased frequency, lungs, bones, and abdomen/peritoneum were the sites of extra-hepatic recurrences, followed by adrenal gland, and brain, without significant differences between LLR and RLR groups (all *p* > 0.05). Two patients had multiple sites (> 1) of extra-hepatic recurrences after LLR while none after RLR (*p* = 0.496). Pattern and distribution of recurrences are detailed in Table 3.

#### Treatment of recurrences

Ten patients out of 51 who developed HCC recurrence (19.6%) underwent salvage-LT [LLR 6 (22.2%) vs RLR 4 (16.7%); *p* = 0.744]. Redo-hepatectomy (either by MILS or open surgery) for HCC recurrence was performed in 8 patients (15.7%) [LLR 1 (3.7%) vs RLR 7 (29.2%); *p* = 0.062] tending to be more used in patients underwent previous RLR. Twenty-seven patients out of 51 (52.9%) with recurrence underwent locoregional treatments [LLR 14 (51.9%) vs RLR 13 (54.2%); *p* = 0.782]. Specifically, ablation (RFA) was the treatment of choice in 16 (31.4%) [LLR 8 (29.6%) vs RLR 8 (33.3%); *p* = 0.767]; followed by TACE in 15 (29.4%) [LLR 7 (25.9%) vs RLR 8 (33.3%); *p* = 0.548]; TARE in 1 (2.0%) [LLR 1 (3.7%) vs none after RLR; *p* = 1.0]. Systemic treatment was used in 12 patients with recurrence (23.5%) [LLR 6 (22.2%) vs RLR 6 (25.0%); *p* = 1.0]; while best supportive care or other unspecified treatments in 6 (11.8%) [LLR 3 (11.1%) vs RLR 3 (12.5%); *p* = 1.0]. Twenty patients (39.2%) received multiple treatments for recurrences [LLR 10 (37.0%) vs RLR 10 (41.7%); p = 0.774]. Details on the treatment of recurrence after PSM are shown in Table 4.

## Discussion

This retrospective study with PSM compared recurrences and survival after RLR and LLR in a cohort of typical patients with HCC of stage BLCL 0-A. We found similar survival (RFS and OS), as well as similar incidence and pattern of HCC recurrence at a median follow-up of 29 months.

Recurrence within 2 years after hepatectomy occurred in 25.7% of the patients and mostly within the liver, which was expected due to the high prevalence of cirrhosis in the population examined [32]. Notably, the proportion of patients developing recurrence within 1 or 2 years after MILS, as well as their OS, did not differ between the two approaches aligning with the other findings (Supplementary Material, Figures S1 and S2). Approximately 4% of patients recurred at the surgical margins and 6% both at the margin and distant within the liver. While recurrence at the surgical margin may be considered a marker of inadequacy of the surgical technique (or the inappropriate choice to perform non-anatomical resections in larger tumors), this low figure was consistent with other studies and may correspond to the fraction of more aggressive HCC with satellite nodules despite the small size [7, 33]. Similarly, distant liver recurrences likely reflect tumor aggressiveness (from the same clone) or the results of the underlying cirrhosis as a precancerous condition.

Another interesting aspect was the use of secondary treatments for recurrences in both groups (transplantation, redo-hepatectomy, locoregional or systemic treatments). Approximately 20% in each group received salvage-LT, suggesting that both techniques preserved LT eligibility. Redo-hepatectomies, however, were performed more often after RLR (29.2%) than LLR (3.7%). The reasons for this difference were probably multifactorial: the choice of treatment in patients with intra-hepatic recurrences includes patient’s condition and liver function (which could be further deteriorated at HCC recurrence), and features of the recurrent-HCC (i.e., single vs multinodular recurrence, superficial vs deep recurrence favoring redo-resection rather than locoregional re-treatments). As these data were not available in the present investigation, this point should be the object of further studies.

For HCC patients with underlying cirrhosis within Milan Criteria, liver transplantation (LT) should be the preferred approach [24, 34]. According to our policy, patients were generally listed for LT upfront in case of multiple HCCs (i.e., 2 or more), or advanced cirrhosis (Child B or more), MELD score > 10, severe portal hypertension with history of variceal bleeding. In other cases (generally single nodules with compensated cirrhosis) or when transplantation was not deemed indicated, patients were considered for liver resection and MILS was privileged [35]. Local ablative therapies were indicated in case of deep lesions or in more fragile patients and/or with deteriorated liver function who were not good candidates for surgery.

More than 85% of resections in this study were non-anatomic, and approximately 15% minor anatomic resections (bi-segmentectomy or less) without any major hepatectomy. Many experts advocate the superiority of anatomic over non-anatomic resections to avoid parenchymal congestion after surgery, to reduce the risk of local recurrence and possibly increase survival [36–43]. However, while its survival benefit remains debated, the counterbalances of anatomic resections (i.e., with extra-hepatic glissonean approach) may be a steep learning curve, longer operative time, higher risk of severe complications and longer hospital stay even in expert hands [44, 45]. The short-term results of MILS in the current analysis confirmed its low impact (i.e., “tip-toe” surgery) in selected cirrhotic patients with contained postoperative morbidity and without mortality. This was in line with a preliminary report by our group [3].

A multicentric registry-based study from Italy evaluated the outcomes of LLR and RLR (non-anatomic resections 66%) in the setting of small (< 3 cm) single HCC in 714 patients, of whom around 30% pre-treated (surgery, locoregional treatments or other), 75% with underlying cirrhosis and 35% with portal hypertension. The study supported the safety and oncologic efficacy of MILS in small HCC. Notably, less than 15% of patients underwent RLR in that study [46].

Three recent studies compared RLR and LLR for HCC focusing on oncologic results. Lim, et al. compared RLR vs 3D-LLR from 3 pioneer centers in MILS (Italy and France) and reported similar RFS and OS (3-years). This study included only 93 patients (49 3D-LLR, 44 RLR) in a 6-year period, cirrhotic patients were only 62%, and there was no matching strategy to address selection bias. More important, no data on recurrences and their treatment (i.e., salvage-LT) was provided [9]. Duong, et al. looked at long-term survival after RLR vs LLR in a large population of patients with stage I HCC (according to AJCC staging system 7th edition) from the National Cancer Database (NCBD). The authors found improved OS in patient treated by robotic approach. However, NCBD lacked data on the rate, cause, and stage of cirrhosis, rate of pre-treated HCCs and modality of pre-treatment, as well as on recurrence and re-treatments, which instead constitute an original point of our investigation [10]. Zhu, et al., conducted a prospective study with PSM from a very large referral center in China. The study included 3 cohorts of patients with BCLC 0-A naïve HCC that underwent open surgery vs LLR vs RLR (56 patients each, after PSM 1:1:1) and found similar OS and RFS between the three approaches. Like in ours, in the study by Zhu, et al. the incidence of cirrhosis was approximately 90% (mostly related to HBV infection), HCCs consisted in a single nodule in > 90% of cases, minor resections were approximately 90% (of which half non-anatomic). The authors also provided detailed description of recurrences and re-treatments. Interestingly, the choice of the surgical approach (RLR vs LLR) was based on patient’s wish, and recurrence profile was slightly different between MILS and OS (more solitary and early-stage recurrences after MILS, relatively easy to manage), finally LT was not mentioned among re-treatment strategies. The study was very well conducted but the transposition of its conclusions in western countries could be questioned [7].

The current study has some limitations. First, the retrospective nature of the analysis may introduce a selection bias between robotic and laparoscopic approaches. However, the selection process for the two surgical techniques was similar, and the two cohorts consisted of similar patients as per baseline features and backgrounds. Moreover, the variables Iwate score (which accounts for tumor location), single vs multiple nodules, and previous treatments were included and well balanced with the PSM, making the influence of potential bias minimal and acceptable in our opinion. The study included the learning curve of both robotic and laparoscopic procedures, another potential source of bias. We thoroughly analyzed our learning curve in a previous study where we showed similar learning phases for the operating surgeons participating in the present investigation [28]. Indeed, in the current analysis, both Iwate scores and R0 rates were similar in RLR and LLR after the PSM, thus substantially mitigating a possible effect of an ongoing learning curve. Last, we included a small population with a relatively short follow-up (median 29 months): some difference in recurrence and survival between RLR and LLR could emerge with a longer follow-up or in a larger population.

Based on our experience and considering the study’s limitations, RLR was non-inferior to LLR in terms of survival outcomes, recurrence rates and patterns, as well as access to secondary treatments in HCC patients with cirrhosis and well-preserved liver function. By addressing gaps in the existing literature (i.e., oncologic outcomes, detailed description of timing, site, and treatment of recurrences) and emphasizing the importance of proper patient selection and surgical technique, the findings of this study contribute to the ongoing discussion surrounding optimal surgical strategies for HCC patients.

## Supplementary Information

Below is the link to the electronic supplementary material.

Supplementary file1 (JPG 252 KB)—Kaplan-Meier curve for Overall Survival (OS) before PSM in patients with recurrence within 1 and 2 years

Supplementary file2 (JPG 253 KB)—Kaplan-Meier curve for Overall Survival (OS) after PSM in patients with recurrence within 1 and 2 years
