# Supplementary figures and images for: Recurrence and survival after robotic vs laparoscopic liver resection in very-early to early-stage (BCLC 0-A) hepatocellular carcinoma
Source: Surg Endosc. 2025 Feb 4;39(3):2116–28. doi: 10.1007/s00464-025-11553-3 (PMC11870908; doi:10.1007/s00464-025-11553-3)

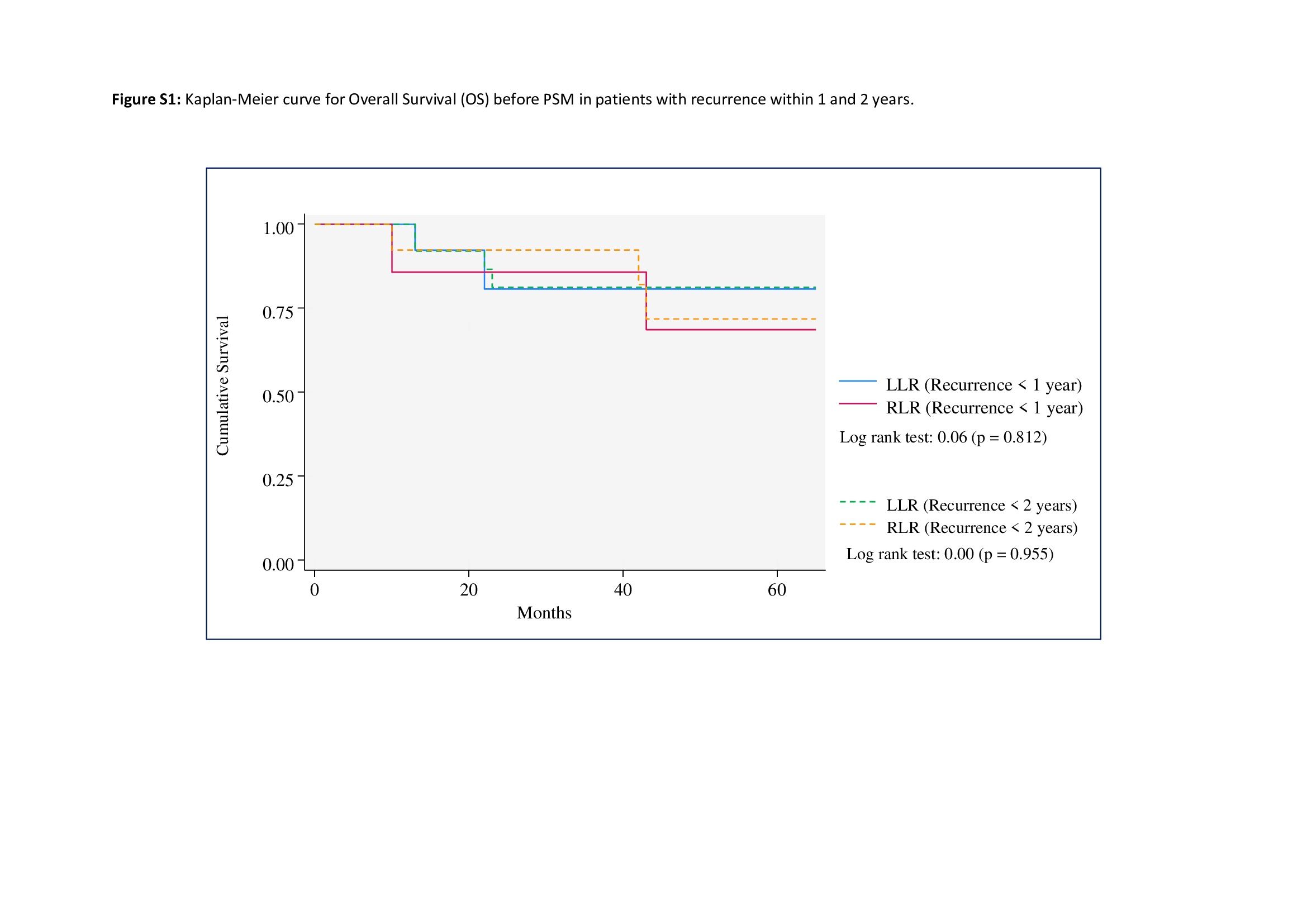

Supplement: Supplementary file 1 — Supplementary file1 (JPG 252 KB)—Kaplan-Meier curve for Overall Survival (OS) before PSM in patients with recurrence within 1 and 2 years [file 464_2025_11553_MOESM1_ESM.jpg]

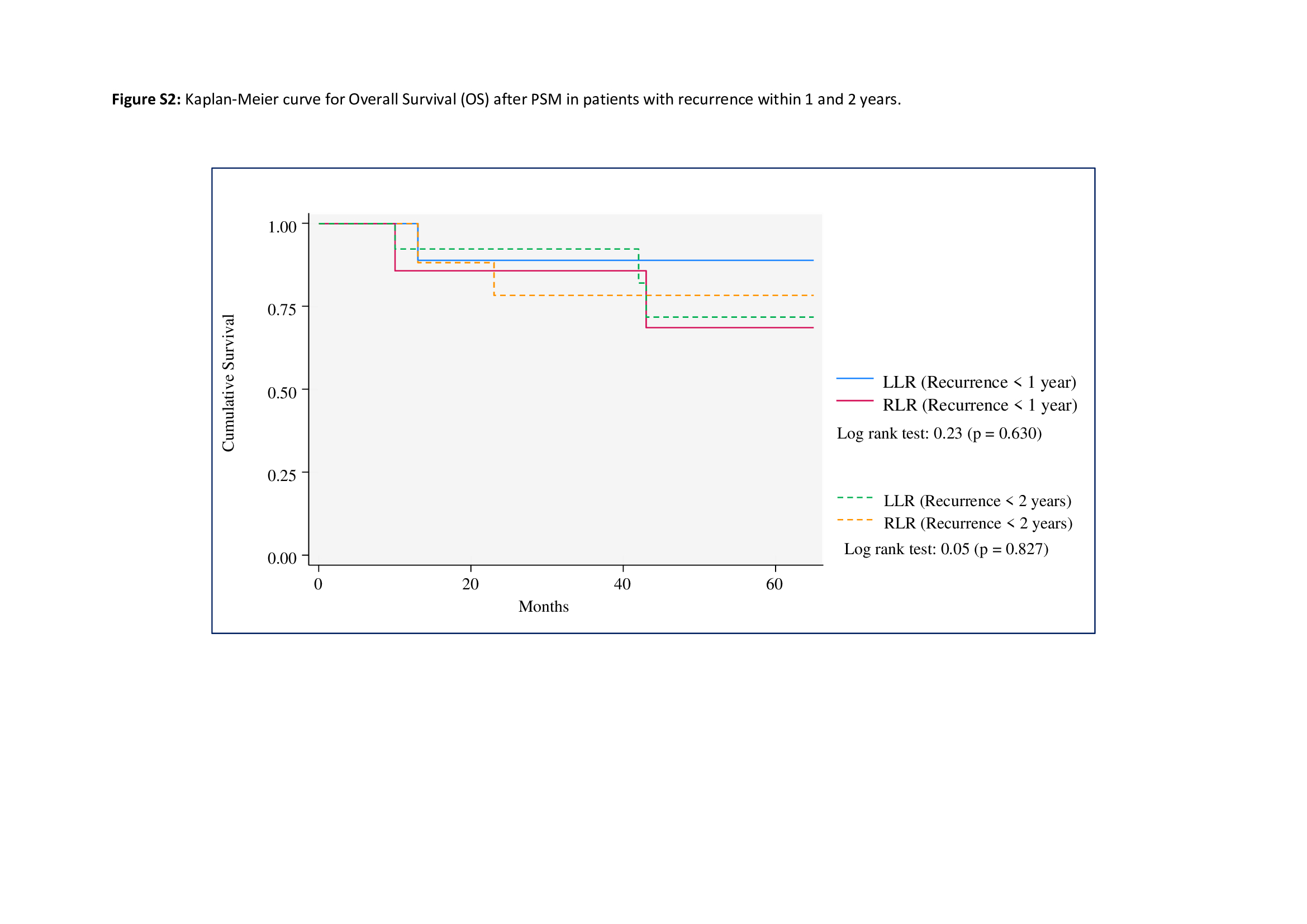

Supplement: Supplementary file 2 — Supplementary file2 (JPG 253 KB)—Kaplan-Meier curve for Overall Survival (OS) after PSM in patients with recurrence within 1 and 2 years [file 464_2025_11553_MOESM2_ESM.jpg]
